# A Probabilistic Approach in the Search Space of the Molecular Distance Geometry Problem

**DOI:** 10.1021/acs.jcim.4c00427

**Published:** 2024-11-13

**Authors:** Rômulo S. Marques, Michael Souza, Fernando Batista, Miguel Gonçalves, Carlile Lavor

**Affiliations:** † Instituto de Matemática, Estatística e Computação Científica, Universidade Estadual de Campinas, Campinas 13083-859, Brazil; ‡ Departamento de Estatística e Matemática Aplicada, Centro de Ciências, Universidade Federal do Ceará, Fortaleza 60020-181, Brazil

## Abstract

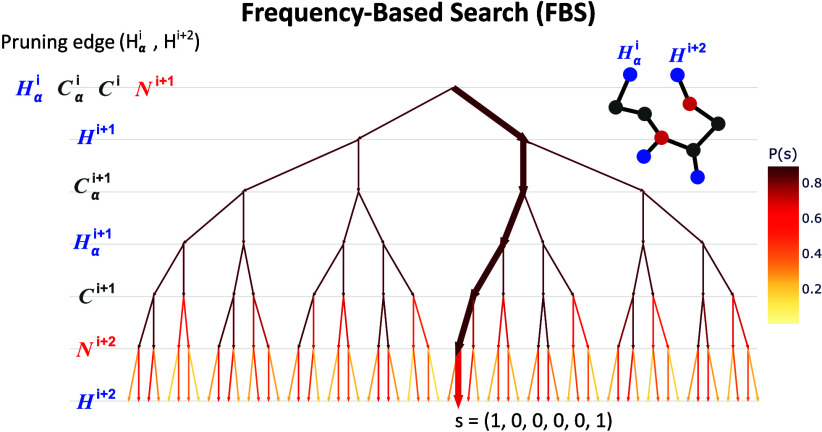

The discovery of the three-dimensional shape of protein molecules using interatomic distance information from nuclear magnetic resonance (NMR) can be modeled as a discretizable molecular distance geometry problem (DMDGP). Due to its combinatorial characteristics, the problem is conventionally solved in the literature as a depth-first search in a binary tree. In this work, we introduce a new search strategy, which we call frequency-based search (FBS), that for the first time utilizes geometric information contained in the protein data bank (PDB). We encode the geometric configurations of 14,382 molecules derived from NMR experiments present in the PDB into binary strings. The obtained results show that the sample space of the binary strings extracted from the PDB does not follow a uniform distribution. Furthermore, we compare the runtime of the symmetry-based build-Up (SBBU) algorithm (the most efficient method in the literature to solve the DMDGP) combined with FBS and the depth-first search (DFS) in finding a solution, ascertaining that FBS performs better in about 70% of the cases.

## Introduction

The determination of the three-dimensional structure of protein molecules represents a profoundly complex challenge within structural biochemistry. This task necessitates a multidisciplinary strategy that melds mathematical modeling, judicious use of computational resources, and experimental data.^1,2^ Among the models developed to tackle this challenge, the discretizable molecular distance geometry problem (DMDGP) utilizes nuclear magnetic resonance (NMR) data to compute the Cartesian coordinates of atoms within the molecule based on distance measurements provided by NMR techniques.^3−5^

In the ideal scenario where the distances between all pairs of atoms in a protein are known, the DMDGP can be solved efficiently in linear time.^6^ However, NMR experiments typically provide only partial and approximate distance data, represented as lower and upper limits, rather than precise values.^7^ A common assumption in the literature is to fix bond lengths and bond angles of the protein molecule whose 3D structure we want to determine,^8^ which despite simplifying the problem, allows us to use a mathematical model (the DMDGP) that exploits the important combinatorial features of the problem, not considered in the continuous approach (see the subsequent sections for further discussion).

The most efficient method proposed in the literature to solve the DMDGP is the symmetry-based build-up (SBBU) algorithm,^9^ which employs a depth-first search (DFS) that explores the search space of the DMDGP, represented as a binary tree.^3^ While DFS is known for its low memory footprint,^10^ it does not incorporate biochemical information about proteins. In this paper, for the first time, we leverage data from the protein data bank (PDB)^11^ to propose an alternative search strategy to DFS.

## The DMDGP Search Tree

The DMDGP is a specific subclass of the molecular distance geometry problem (MDGP),^12^ which is defined as follows.

Given a weighted undirected graph *G* = (*V*, *E*, *d*), where *V* represents the set of atoms in the molecule and *E* is the set of atom pairs with known distances given by , solving the MDGP involves finding a function  such that
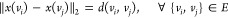
1

The DMDGP is an MDGP with a particular ordering of the vertices of  and specific conditions on the known distances. Specifically, in the DMDGP, each vertex  (for ) must be connected to its three immediate predecessors  with known distances. The conditions for the ordering  are as follows (since the DMDGP can also be defined in other dimensions *k*, represented as , for notational simplicity we simply write DMDGP for the particular case ):

H 1 : The first three vertices form a clique and satisfy eq 1.
H 2 : For each vertex  with , we have .

A simplified notation is used where the vertices  are represented by their indices  only. Thus,  represents the coordinates of the vertex  and  represents the distance between vertices  and .

To eliminate solutions obtained merely by the rotations and translations of a given solution, the positions of the first three atoms can be fixed as follows:
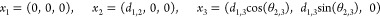
forming the vertices of a triangle with sides , where θ_2,3_ is the angle at  determined by the Law of Cosines.

**Figure 1:**
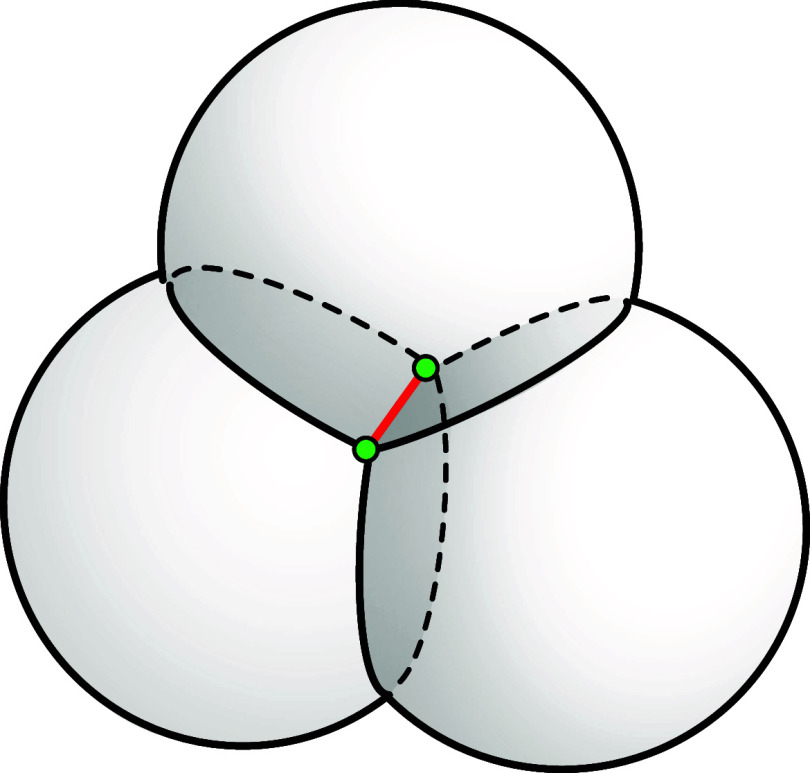
Two points (green) are in the intersection of three spheres.

Subsequently, in an iterative construction approach to solving the DMDGP, under hypothesis H_2_ (we say that the immediate predecessors  serve as reference vertices for *v*_*i*_ and that the distances , ,  are called discretization distances,^13^ we can solve the following system:
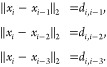
2Each of these constraints defines a sphere centered at one of the immediate predecessors, with a radius equal to the distance between this center and point . Therefore,  must lie in the intersection of these three spheres. Assuming a solution exists for the DMDGP and that the points  are not collinear, the intersection of these three spheres will consist of at most two points (see Figure 1).

Through this constructive procedure, once atoms  are fixed, atom  will have two possible positions,  or  in , obtained from system (2). Once  is chosen from the two possibilities, we can continue the process by fixing point .

**Figure 2:**
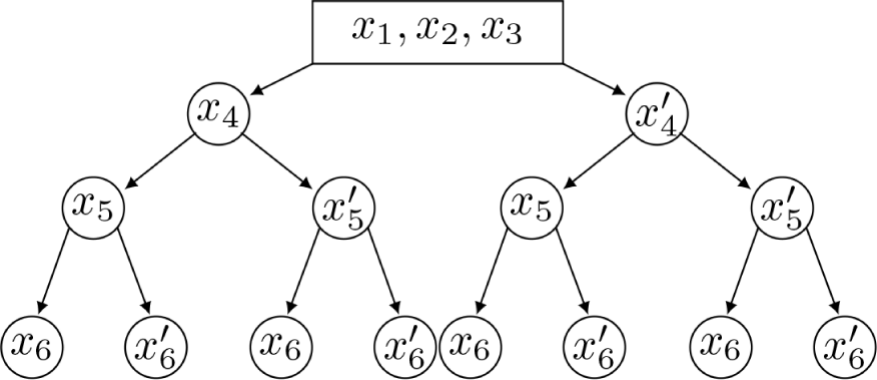
Binary tree associated with a DMDGP instance with six atoms.

A natural representation for all possible configurations is a binary tree, where  are represented as a single root node (since they are fixed) and their two children represent the two possibilities for , and for each of these, we have two possibilities for , and so on (see Figure 2).

**Figure 3:**
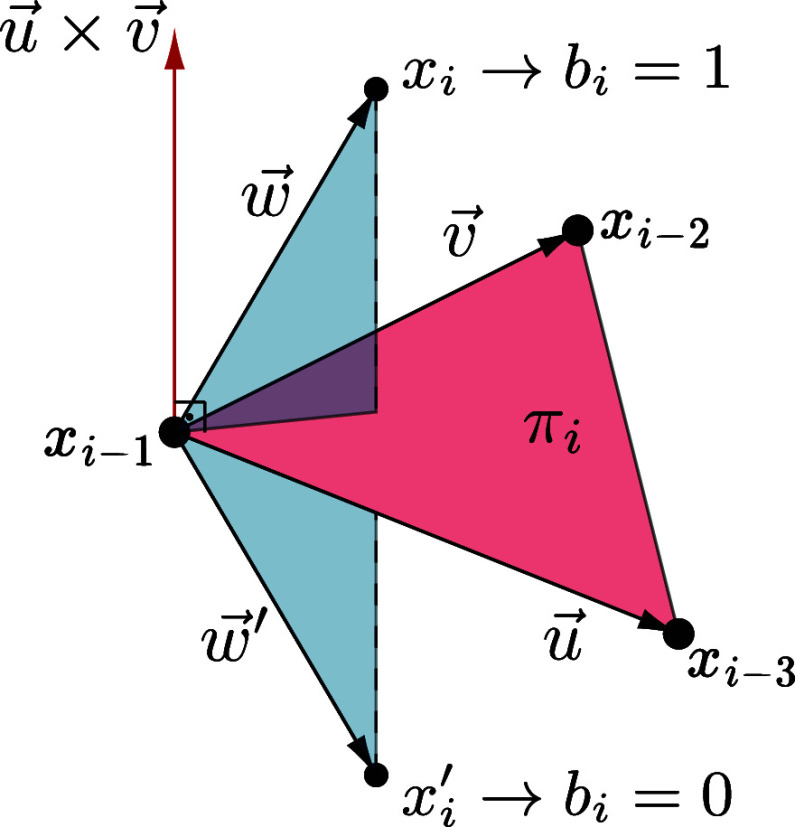
Plane π_*i*_ and the positions *x*_*i*_ and  associated with orientations 0 and 1. The possible immersion points (in ) for the vertex *v*_*i*_ are *x*_*i*_ and : the point “above” the plane π_*i*_ is *x*_*i*_ and has orientation *b*_*i*_ = 1; the point “below” π_*i*_ is  and has orientation *b*_*i*_ = 0.

If we designate the left node as child 0 and the right one as child 1, we can also map the relative position of each realization of  with respect to the plane π_*i*_ defined by , which are the immediate predecessors of . Specifically, we can define the vectors
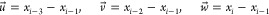
and assign orientation 0 if , and 1 otherwise (see Figure 3).

When additional distances are known (namely *d*_*ij*_, where *j* is not one of the immediate predecessors of atom *i*), the viable configurations are reduced by the presence of the additional constraint . These additional distances are referred to as pruning distances because they make the branches in the DMDGP binary tree infeasible.^13^

Together with the definition of the DMDGP, the branch-and-prune (BP) algorithm^13−15^ was designed to solve the problem by intelligently traversing the DMDGP search tree. Using the pruning constraints, it prunes branches that are identified as infeasible, thus eliminating the need to explore the entire tree.

Since there exists an algorithm that outperforms BP (the SBBU algorithm,^9^ as we mentioned in the introduction, we will use it to compare two different approaches to explore the DMDGP binary tree (we will give more details about SBBU in Extracting binary sequences).

The SBBU algorithm was originally developed to use a depth-first search (DFS) strategy that arbitrarily favors the 0 nodes of the binary tree. For example, in a binary tree of length two, the explored paths would be 00, 01, 10, 11.

DFS is a fundamental algorithm used in tree traversal,^10^ characterized by exploring a branch as deeply as possible before backtracking to explore other branches. While it always finds solutions in finite trees, DFS is notable for its memory efficiency, as it needs to store only a stack of nodes on the current path from the root node. However, it is important to note that DFS does not guarantee the shortest path to the solution.

In contrast to DFS, we propose a best-first search strategy called frequency-based search (FBS), an algorithm that traverses the tree by selecting which path to follow based on an evaluation function that estimates which nodes are most likely to lead to a solution.

### An FBS Approach Defined by the PDB

The protein data bank (PDB) is a crucial resource for scientific advancement, containing over one terabyte of structural data for proteins, DNA, and RNA. The archive grows by nearly 10% per year and facilitates over 5 million structure data file downloads daily.^16^ There are 194,992 protein-related entries in the PDB, of which 14,382 were obtained through NMR.^17^

To assemble our data repository, we selected all protein structures derived from NMR experiments present in the PDB, considering the relevance of such a technique to the scope of our research. From the first model of each selected PDB file, we extracted the following information for the backbone atoms of the protein in question: its unique index, its name (*N*, *C*_α_, *C*, *H*, *H*_α_), and its coordinates in , as well as the index and three-letter abbreviation of the residue to which it belongs.

It is important to highlight that some PDB files do not completely describe the protein backbone. For example, there are files in which some residues are missing, and in others, some atoms are missing. We also chose to remove proline and glycine residues, as these amino acids exhibit unique geometric characteristics and are often studied separately in the literature.^18,19^

We refer to the stretches of the backbone formed by contiguous residues after the removal of prolines and glycines as protein segments. Thus, associated with each PDB file, we generated several files, one for each protein segment. The total number of protein segments obtained from all the NMR files in the PDB was 73,675.

### A DMDGP Order to Be Used in the FBS

The first step in establishing an FBS strategy that incorporates PDB data is to determine a DMDGP order for the atoms in the backbone. We present a DMDGP order that utilizes the lengths of covalent bonds, bond angles, and the geometric properties of peptide planes (in order to maintain the DMDGP symmetry properties,^20,21^ some of the atoms must be repeated in the order.^22^

**Figure 4:**
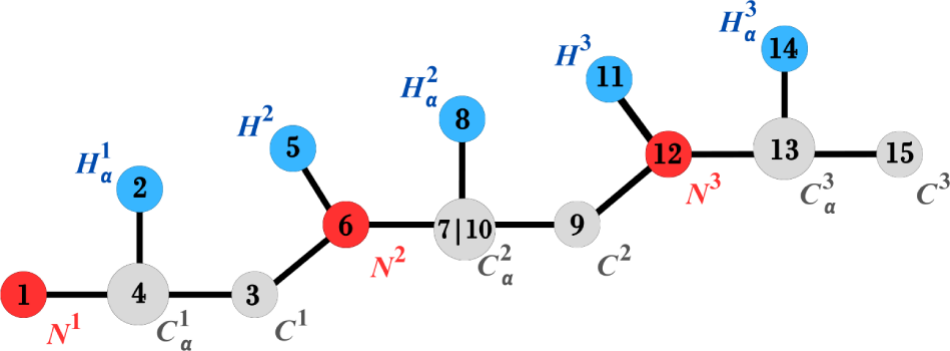
Order ρ of a 3-residue peptide backbone. The number inside each circle represents the position of the respective atom in the order of ρ. The repetition of  is the 10th element of ρ.

In the context of protein geometry, it is considered that bond lengths and bond angles are fixed, despite the natural internal motions of proteins. This assumption is known as the rigid geometry hypothesis.^23^ Consequently, the distances between atoms connected by one or two covalent bonds are known. In addition to this distance information, it is well established in the biological literature that the atoms “around” a peptide bond belong to the same plane, implying that all distances between these atoms are also known.^24^ Since the peptide bond connects the carboxyl carbon of the *i*-th residue (*C*^*i*^) to the amine nitrogen of the (*i*+1)-th residue (), the atoms in the *i*-th peptide plane are , *C*^*i*^, ,  (see Figure 4). We also consider that the distances between *H*^*i*^, , and ,  can be detected by NMR.^25−27^

Based on these properties, we use the following DMDGP order for atoms in the protein backbone:
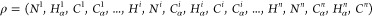
3Note that for each intermediary residue we repeat the *C*_α_ after the occurrence of the *C* atom.

Figure 4 illustrates the order ρ for a three-residue peptide (the numbers inside the circles indicate the index of the atoms in the order) and Table 1 gives more information about ρ.

**Table 1:** Reference Atoms in Order ρ^a^

Order	Atom	Predecessor Positions			Predecessor Atoms		
1	*N*^1^						
2							
3	*C*^1^						
4		3	2	1	*C*^1^		*N*^1^
5	*H*^2^	4	3	2		*C*^1^	
6	*N*^2^	5	4	3	*H*^2^		*C*^1^
7		6	5	4	*N*^2^	*H*^2^	
8		7	6	5		*N*^2^	*H*^2^
9	*C*^2^	8	7	6			*N*^2^
10		9	8	7	*C*^2^		
							
	*H*^*i*^						
	*N*^*i*^				*H*^*i*^		
					*N*^*i*^	*H*^*i*^	
						*N*^*i*^	*H*^*i*^
	*C*^*i*^						*N*^*i*^
					*C*^*i*^		
							
	*H*^*n*^						
	*N*^*n*^				*H*^*n*^		
					*N*^*n*^	*H*^*n*^	
						*N*^*n*^	*H*^*n*^
	*C*^*n*^						*N*^*n*^

a For each atom in ρ, the first column displays its position; the second column displays its symbol; the third column displays the positions in ρ of its reference atoms; and the fourth column displays the symbols of its reference atoms.

### Binary Representation for the Protein Backbone

There are different representations for proteins. In principle, they can be represented by strings formed from 23 characters, each representing one of the possible amino acid residues. This is a unique representation but is not geometric. Another possible representation is a list of 3D coordinates defining the location of each atom composing the protein.

Since the solution space of a DMDGP can be organized as a binary tree, a solution can be represented as a binary string that incorporates geometric information. Formally, following the ordering ρ (given in (3)), each atom at position *x*_*i*_ can be associated with a bit *b*_*i*_ based on its relative position to the plane formed by its reference atoms. Thus, the Cartesian coordinates of each protein segment can be mapped, in a one-to-one relationship, to a *binary sequence*.

Given that the first three atoms of ρ are easily fixed in  and have no reference atoms (see Figure 4), we consider *b*_1_ = *b*_2_ = *b*_3_ = 0, without loss of generality (although a repeated vertex *v*_*i*_ can have reference atoms, the calculation of *x*_*i*_ results in a point identical to the first occurrency of the atom of *v*_*i*_. In this case, consider it has a fixed ). We can notice that for the fourth atom of ρ, the two possible positions for immersing it in  (resulting from the intersection of the spheres associated with *x*_1_, *x*_2_, *x*_3_) are always feasible, as *v*_4_ never has a fourth preceding neighbor. This implies that for every solution *x* where *b*_4_ = 0, there exists another solution *x*^′^, where *b*_4_ = 1, obtainable by reflecting *x* with respect to the plane π_4_ that passes through points *x*_1_, *x*_2_, *x*_3_.

As a direct consequence of this unique characteristic of *v*_4_, we know that the binary representation of *x*^′^ is the total inversion of the bits of *b* from the fourth position onward (except for binary variables of repeated vertices). Therefore, to reduce the representation of structures obtained in this manner, we normalize our data set by inverting all binary representations where *b*_4_ = 1. With this choice, the binary representations in our database have the format . We adopt the reduced representation , removing the first four fixed bits.

### Extracting Binary Sequences from the PDB

The SBBU algorithm solves one pruning constraint at a time. The constraints are of the form  when represented in Cartesian coordinates, but each of these constraints has a binary version, , where  is the coordinate of *x*_*i*_ as a function of the binary representation *b*. A change in the bits *b*_*k*_, for , may alter the coordinates of  but does not affect the distance between them. Therefore, only the bits  are “relevant” (we will use this term as a definition) for satisfying the associated constraint.

In SBBU,^9^ the pruning edges are ordered and the associated constraints are solved sequentially. When a constraint is *coupled* with a preceding one, that is, if it shares relevant binary variables, we can utilize an important result from the DMDGP symmetries.^28^ This result states that if  is a viable binary sequence for , then the only other viable binary sequence for this constraint is the complete flip of . This means that when we have coupled constraints, we can take advantage of part of the solution already found as well as being part of the other constraint (of course, considering also its complete flip).

When a constraint does not share relevant binary variables, we refer to it as an independent constraint, and in such cases, SBBU must perform an exhaustive search for viable binary sequences.

The symmetric properties of the DMDGP^20^ allow us to derive all solutions from a single solution. Consequently, it is unnecessary to determine the PDB conformation as we can directly utilize the first solution found by SBBU. Therefore, when constructing our database, instead of extracting the binary sequences of independent constraints directly from the PDB, we solve the associated DMDGP instances and extract the binary sequences from the first solution that SBBU identifies for each independent constraint.

As the next step, we organize the extracted binary sequences by their first and last atoms as well as their lengths. For example, consider *d*_2,11_, an independent pruning distance related to the molecule illustrated in Figure 4. The atomic names of vertices 2 and 11 are *H*_α_ and *H*, respectively, and the length of the segment  is 10. Thus, we classify the binary solution associated with *d*_2,11_ into the group .

Our complete data set, comprising 73,675 three-dimensional configurations and their respective binary codings, can be automatically generated using the Python script available in the repository https://github.com/romulomarques/proteinGeometryData(accessed on 30 May, 2024). Additionally, this repository includes a .csv file containing the PDB IDs of all of the instances we downloaded.

### The FBS in the SBBU Algorithm

As we already mentioned, the original version of the SBBU algorithm utilizes a DFS strategy. However, we can employ a more sophisticated search method that takes into account the probability distributions of viable binary sequences associated with solutions of DMDGP instances.

The new search strategy, FBS, is based on identifying a viable path by analyzing the frequency of binary sequences associated with specific independent edges. More specifically, we group the viable binary sequences of independent edges into categories such as , as mentioned above. For example, another group utilized is , which contains all constraints , where atom *i* is carbon and atom *i* + 4 is α-carbon. Subsequently, we sort the sequences within each group, prioritizing the most probable ones (i.e., those with the highest frequency).

**Figure 5:**
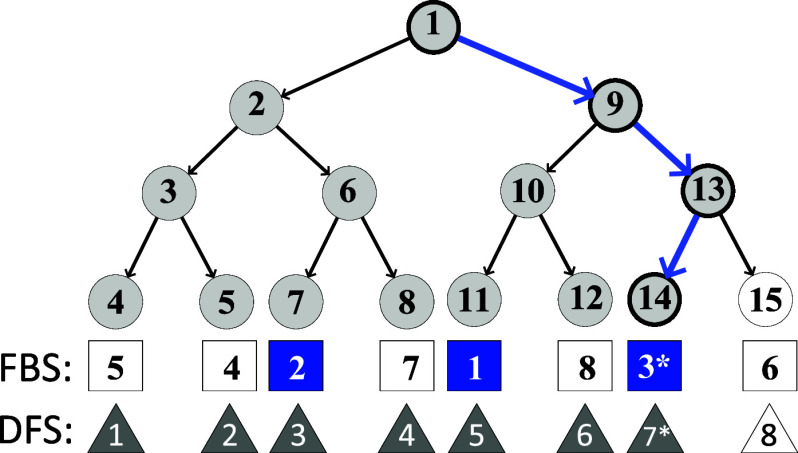
Binary tree nodes visited by DFS and FBS. The number in each node represents its position in the DFS visiting order. The number within a square at the bottom of each tree leaf indicates the position of the respective root–leaf path in the FBS. Similarly, the number within a triangle denotes the position of the path in DFS. The blue arcs highlight the root–leaf path corresponding to the solution. Blue squares and gray triangles illustrate which root–leaf paths are explored, respectively, by FBS and DFS until a solution is found (marked with *).

The optimal tree search strategy minimizes the number of visited nodes. In FBS, paths associated with the most frequent sequences are tested first. Assuming the binary solution is , each alternative path is tested separately: if the position of *b* in the FBS order is *k*_*b*_, then *k*_*b*_ paths of length *n* must be tested, resulting in a total cost of
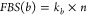
4DFS operates in a similar manner within the SBBU algorithm. However, tree paths are ordered from the leftmost to the rightmost. If the position of *b* in this default ordering is *l*_*b*_, the number of visited nodes is given by
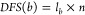
5Figure 5 illustrates a binary tree of height four, composed of nodes numbered from 1 to 15. Below the tree representation, squares indicate the FBS ordering of the eight paths from the root to the leaves (ordering learned from the PDB), and triangles indicate the DFS default ordering. Suppose that the path (1, 9, 13, 14), highlighted in blue, represents the binary solution. Additionally, assume that *b* is in the third position in the FBS order. Under these assumptions, only the rightmost path would not be evaluated by DFS in the search for solution *b*, while the FBS algorithm would evaluate three paths of length four, involving a total of 12 nodes: (1, 9, 10, 11), (1, 2, 6, 7), and (1, 9, 13, 14).

Applying eqs 5 and 4, we obtain
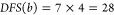
and
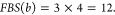


Therefore, FBS can reduce the number of visited nodes compared with DFS by prioritizing paths with frequent sequences. In the next section, we present a comparative analysis of SBBU combined with FBS and DFS to demonstrate this performance difference.

## Computational Results

In this section, we present a descriptive statistical analysis of the binary sequences processed from our PDB data set. We highlight that the distribution of these sequences is not uniform. Furthermore, we randomly split the protein segment instances into training and test sets with an 80%–20% ratio, respectively. The training set provides the binary sequence frequency information that is used in the FBS strategy. Then, we compare the performances of DFS and FBS in terms of the execution time of the SBBU algorithm.

The SBBU algorithm was implemented in C++, with output processing handled in Python. The experiments were run on a machine equipped with a 13th Gen Intel(R) Core(TM) i9–13900H processor (2.60 GHz), 16GB RAM, and a Linux operating system.

Although there may be independent pruning edges of other types, we restrict our analysis to sequences of types , , and  for two reasons: (i) the SBBU algorithm works by identifying viable binary sequences and then assembling them together. Hence, the algorithm underperforms when searching for the solution of independent constraints involving many binary variables because the search space size is an exponential function of the binary sequence length; (ii) the frequency of larger sequences is relatively low compared to the number of sequences of smaller lengths.

In Table 2, we observe that for all edge types, the fraction of observed binary sequences (*k*/*k*_max_) is equal to 1. This indicates that every possible binary sequence of the specified length is present in our data set. Additionally, the ratio of Count to *k*_max_ varies significantly across different edge types, reflecting the differing frequencies of each sequence. For instance, the edge type HA-7-HA with a length of 4 has a Count/*k*_max_ ratio of 25,225.25. If we had a uniform distribution of sequences, we would expect each unique sequence to be observed 25,000 times on average. In contrast, the C-5-CA edge type with a length of 2 has a ratio of 2913.00, indicating a lower average frequency per sequence.

**Table 2:** Frequency Information on Binary Sequences for Each Edge Type^a^

Edge Type	Length	Count	*k*	*k*_max_	*k*/*k*_max_	Count/*k*_max_
HA-10-H	7	78,943	64	64	1.00	1,233.48
HA-7-HA	4	201,802	8	8	1.00	25,225.25
C-5-CA	2	5,826	2	2	1.00	2,913.00

a The second column shows the binary sequence lengths (remember that the first three bits are already fixed); the third column shows the number of binary sequences processed from PDB; the fourth column shows the number of unique binary sequences collected; the fifth column shows the number of unique binary sequences that are mathematically possible to exist; the sixth and seventh columns represent the ratios of the third and fourth columns to the fifth column, respectively.

For each edge type, the FBS strategy sorts the binary sequences in descending order, with respect to their occurrence probabilities. In the following, we normalize the order indices by dividing them by the total number of sequences of each length. As a result, the normalized indices have values in the range (0, 1]. That is, if we have ordered sequences *s*_1_, *s*_2_, and *s*_3_, then the normalized indices would be 1/3, 2/3, and 1, respectively.

**Figure 6:**
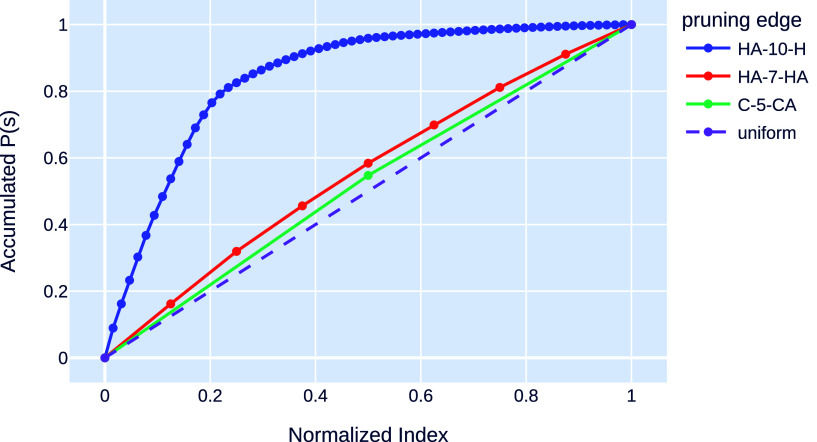
Accumulated probability of the occurrence of the protein binary sequences. For each type of independent pruning distance, each binary sequence configuration processed from PDB is mapped, in descending order of probabilities, to an index in the interval (0, 1]. The dashed line represents the accumulated probabilities of a uniform distribution.

Figure 6 presents the accumulated probabilities for sequences of each edge type using the same normalized indices. It is observable that edges of the types HA-7-HA and C-5-CA have a distribution close to the uniform distribution. On the other hand, the edge type HA-10-H has a distribution with a sharp peak at the beginning, followed by a rapid decline. Therefore, for the edge type HA-10-H, a small number of sequences concentrate most of the probability mass. By giving priority to the most frequent sequences, we can significantly reduce the computational cost to find a feasible solution to edges in the HA-10-H class.

In order to compare the performance of DFS and FBS, for each instance of the problem, we measured the execution time of the SBBU algorithm to solve each edge, for each edge type, and also the total execution time.

**Figure 7:**
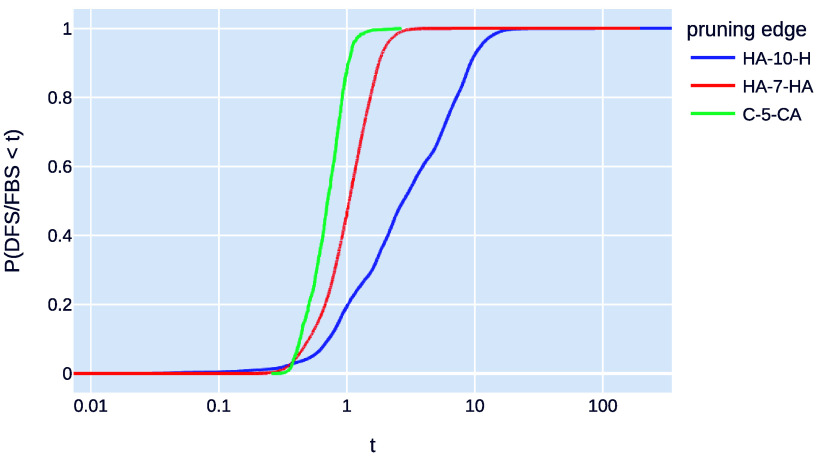
Probability that the time speedup (the ratio of SBBU-DFS time to SBBU-FBS time) for binary sequences of different types of independent pruning distances is less than α, where α can go up to 100. If α is greater than 1, it means that FBS performs better than DFS.

Figure 7 shows the accumulated probability distribution for the relative time (speedup) of DFS over FBS for each edge type. In this context, a speedup greater than 1 indicates the superior performance of the FBS algorithm compared to DFS. As can be seen, for the edge type HA-10-H, FBS is better than DFS in approximately 80% of the cases. For the edge type HA-7-HA, FBS is better than DFS in approximately 60% of the cases. For edge type C-5-CA, FBS is worse than DFS in approximately 90% of the cases.

**Figure 8:**
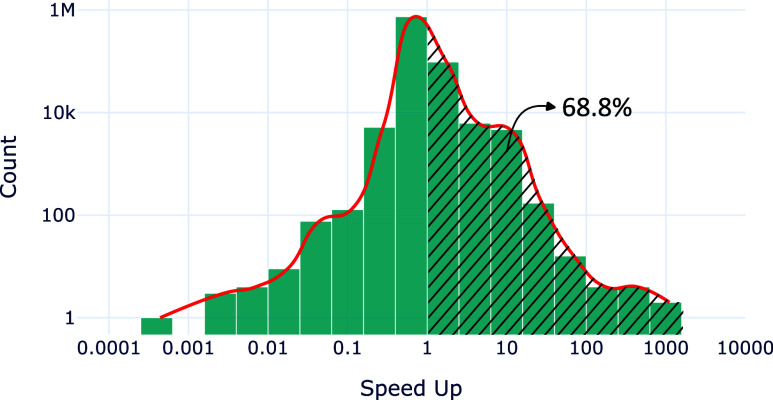
Histogram of the total time speedup (the ratio of the time that SBBU-DFS spent to solve an instance completely to the respective SBBU-FBS time). The hatched area indicates the number of instances where FBS is better than DFS, which corresponds to 68.8% of all of the tested proteins.

The poor performance of FBS in the C-5-CA class does not affect its overall performance. In fact, Figure 8 shows that the speedup of solving the instances completely is greater than 1 in approximately 68.8% of the cases, which means that FBS is better in almost 70% of the tested problems. This is also reinforced by the right-skewed distribution.

**Figure 9:**
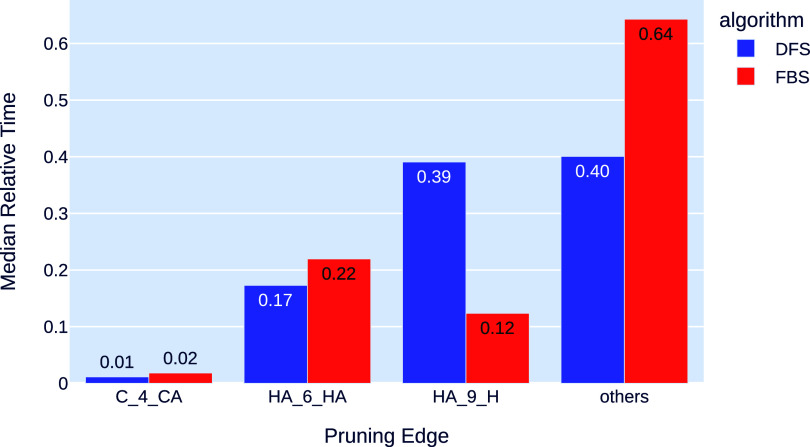
Median relative time of each type of independent pruning distance when the problems are solved using DFS (blue) and FBS (in red). The relative time of a pruning distance type is the fraction of the total time spent solving all instances that corresponds to that type of distance. The “others” column corresponds to the relative time of all dependent pruning distances combined.

Figure 9 shows the average portion of the time spent by the SBBU algorithm in each edge class. On average, for DFS, the edge types HA-10-H and HA-7-HA correspond to 39% and 17% of the total time, respectively, while the time spent solving the C-5-CA edge type is almost negligible. On the other hand, the FBS strategy reduces the portion of time spent solving HA-10-H edges from 39% to 12%. The efficiency of FBS observed in Figure 8 can be explained by leveraging the edge types HA-10-H and HA-7-HA, as shown in Figure 7, and their relative importance in the total time. For FBS, the last column of Figure 9 shows an increase in the relative time spent solving dependent pruning edges as a consequence of the reduced time spent on HA-7-HA and, more significantly, on HA-10-H edges.

Our experiments demonstrate a clear advantage of the FBS method over the traditional DFS, thereby fulfilling the primary objective of this paper. Although the binary sequences extracted and analyzed from the PDB represent a specific subset of proteins, particularly those characterized by using NMR techniques, the data provide a robust foundation for our analysis and support the effectiveness of the FBS method within this context.

## Conclusions

This study represents a pioneering effort in integrating data from the protein data bank (PDB) to address the discretizable molecular distance geometry problem (DMDGP), a central model in protein structure determination using nuclear magnetic resonance (NMR) data.

Our methodology exploits the combinatorial structure of the DMDGP, where the solution space is organized as a binary tree. By developing a binary string representation for protein backbone atomic coordinates, we identified nonuniform frequency patterns. This discovery led to the creation of the frequency-based search (FBS) method, which utilizes these patterns to enhance the search efficiency within the DMDGP solution space.

We incorporated FBS into the symmetry-based build-up (SBBU) method, the current state-of-the-art approach for solving DMDGP instances. Replacing the traditional depth-first search (DFS) with FBS in the SBBU framework resulted in an efficiency improvement in approximately 70% of tested instances, as evidenced by reduced computational time. This improvement is due to a significant decrease in the number of nodes traversed. Unlike DFS, FBS employs a data-driven strategy based on PDB-derived patterns, increasing the likelihood of identifying viable conformations and enhancing the search efficacy.

The results indicate that future research should focus on adapting FBS to other variants of the distance geometry problem (DGP)^29,30^ and expanding its application to additional structure determination techniques.

## Data Availability

The codes provided by the authors at https://github.com/romulomarques/proteinGeometryData (accessed on 30 May 2024) progressively generates all files and folders used in this research. However, since some of these folders occupy a significant amount of memory, such as the folder containing the files downloaded from the PDB, which amounts to 25 Gigabytes, the authors have decided to make available in the GitHub repository the folder containing the data of protein segments, which is the necessary information to actually reproduce the experiment. In addition, in the same repository, the authors provide a .csv file that lists the PDB IDs of all the downloaded 3D structures. This Github project also provides the C++ implementation of the SBBU algorithm with both of DFS and FBS searches, and the Python codes that generate the graphics of the paper.
